# Transepidermal water loss (TEWL): Environment and pollution—A systematic review

**DOI:** 10.1002/ski2.104

**Published:** 2022-02-25

**Authors:** Maxwell Green, Nadia Kashetsky, Aileen Feschuk, Howard I. Maibach

**Affiliations:** ^1^ Tulane University School of Medicine New Orleans Louisiana USA; ^2^ Faculty of Medicine Memorial University of Newfoundland St John's Newfoundland & Labrador Canada; ^3^ Department of Dermatology University of California San Francisco San Francisco California USA

## Abstract

**Introduction:**

Transepidermal water loss (TEWL) is an objective measurement of skin integrity measured as the amount of water lost across the stratum corneum. TEWL varies greatly across variables such as age and anatomic location, and disruptions in the skin barrier have been linked to inflammatory dermatoses such as psoriasis and atopic dermatitis. Impact of environmental conditions and pollution on TEWL has yet to be determined. Accordingly, this review summarizes effects of environmental conditions and pollution on TEWL.

**Methods:**

A comprehensive literature search was performed using Embase, PubMed, and Web of Science to find human studies that provided data on environmental conditions and/or pollution and TEWL.

**Results:**

In total, 15 studies were included, with 11 studies examining environmental and seasonal conditions on TEWL and four examining pollution. All studies examining pollution showed increased TEWL in people exposed to particulate matter or NO2. Contradictory results were found on the effects of season and climate across the 11 studies, with no consensus reached.

**Conclusion:**

Exposure to pollution is reported to cause increases in TEWL, likely through free radical damage. Significant discrepancies exist among current literature as to the effects of season and climate on TEWL. There is a need to continue examining environmental variables other than temperature and relative humidity, such as atmospheric and steam pressure, that may impact TEWL.

## Graphical abstract

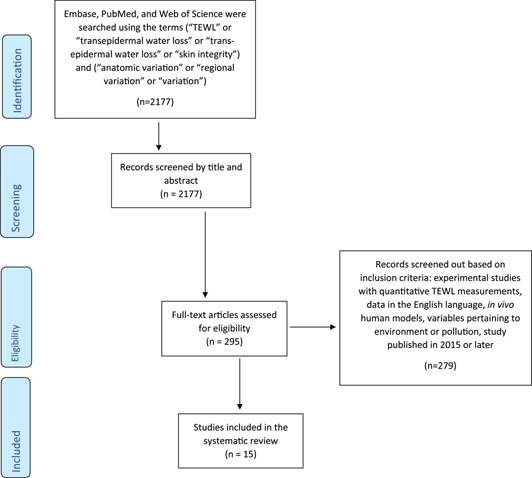

1

**What is already known about this topic?**

- Increases in TEWL have been linked to inflammatory dermatoses such as psoriasis and atopic dermatitis.
- Factors such as age and anatomic location have been linked to variation in TEWL values across individuals.

**What does this study add?**

- This review summarises how both environment and pollution may impact TEWL values across individuals.

## 2 INTRODUCTION

Transepidemal water loss (TEWL) is a measurement that represents the amount of water that escapes from the stratum corneum per area of skin and has historically been used as a reflection of skin water barrier integrity. TEWL measurements are made by placing a probe at the surface of the skin that is able to measure any changes in water vapour density across a determined area of skin over time by sensing changes in local humidity above ambient humidity values.^1^ Disruptions in the skin barrier have been linked to increased TEWL values in dermatologic diseases such as psoriasis and atopic dermatitis.^2^ TEWL varies greatly between individuals and across anatomical locations as described extensively by Akdeniz et al.^3^ Many factors likely contribute to anatomic variation in TEWL values, but increased sweat gland activity at locations such as the forehead compared to the forearm lead to increases in water vapour and TEWL measurements.^4^ Additionally, the systematic review by Peer et al.^5^ summarised additional factors that may impact TEWL and suggested that age and skin surface temperature may impact TEWL. Given such great variation, it is important to avoid adding to this variation by minimising measurement variation in experimental settings.

An additional factor not yet summarised is the impact of climate on TEWL. Imhof et al.^6^ describe how the microclimate between the skin surface and measurement device contribute greatly to measurement accuracy, with rate of evaporation due to skin surface temperature contributing to potential error in measurement without proper calibration. In addition, the temperature of the probe itself can contribute to TEWL values, with higher TEWL values observed with a higher temperature probe.^7^ The temperature and climate conditions at time of measurement are important variables that influence TEWL readings, so it is important to consider how chronic exposure to differing climate conditions in humans may affect skin integrity and TEWL values.

Discrepancies exist amongst the literature as to how great an affect environmental conditions such as temperature and relative humidity have on TEWL. Using a climatic chamber to control conditions, it was found that TEWL significantly increased with increasing temperature and decreased with increasing relative humidity.^8^ This may explain why TEWL values have often been shown to be higher during summer months (higher temperatures) with skin hydration greatly improved, but seasonal variation in TEWL varies greatly by anatomic location.^9^ However, other studies have found contradictory results showing TEWL values to be higher in the nasolabial region during cooler winter months compared to autumn.^10^ Thus, it is important to review the effects of climate on TEWL to understand such contradictions.

In addition to climate conditions like temperature and humidity, chronic exposure to air pollution may also contribute to skin barrier disruption. Air pollution and particulate matter cause damage to the epithelial barrier through oxidation of reactive oxygen species.^11^ Pollution may also worsen dermatologic conditions such as atopic dermatitis through this oxidative barrier disruption and immune system activation cascades.^12^ These mechanisms of skin integrity damage are an important consideration for those living in industrialised regions, and in combination with climate conditions, it may help identify individuals at higher risk for transient increases in TEWL based on geographic location. Thus, our goal is to add to the literature on factors influencing TEWL outlined by Akdeniz et al.^3^ and Honari and Maibach^13^ and summarise recent literature on how climate and pollution affect TEWL in humans.

## 3 METHODS

**FIGURE 1:**
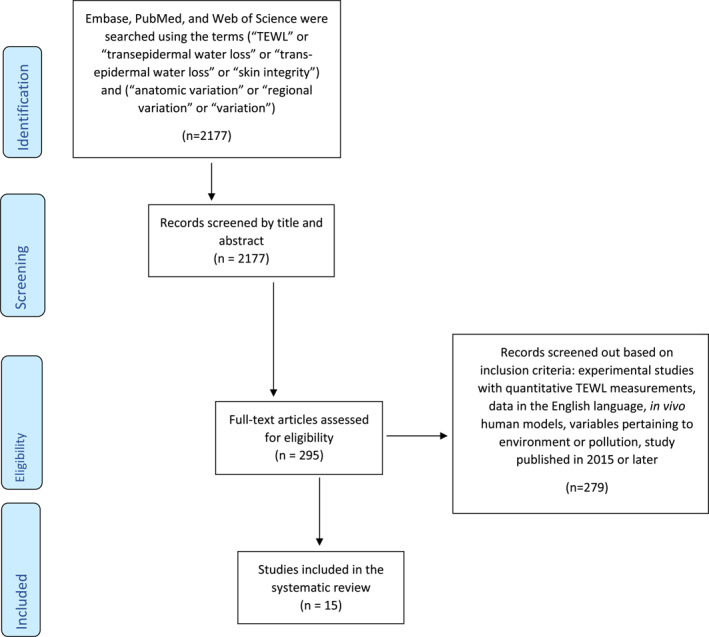
Flow diagram of the literature search using the Preferred Reporting Items for Systematic Reviews and Meta‐Analyses (PRISMA) guidelines. Adapted from http://prisma‐statement.org

The Preferred Reporting Items for Systematic Reviews and Meta‐Analysis (PRISMA) were used to guide the methodology and reporting (Figure 1).^14^ In September of 2021, a comprehensive literature search was performed using Embase, PubMed, and Web of Science using the terms (‘TEWL’ or ‘transepidermal water loss’ or ‘trans‐epidermal water loss’) and (‘anatomic variation’ or ‘regional variation’ or ‘variation’). Only studies after 2015 were included to add to the existing information presented in previous literature such as that done by Akdeniz et al.^3^ and Honari and Maibach.^13^ No geographic or language restrictions were employed. The final inclusion criteria included studies: (1) with quantitative data analysing environmental factors or pollution on TEWL, (2) published after 2015, (3) using in vivo human models.

Title and abstract were used as a first pass to screen articles by two researchers (Maxwell Green and Howard I. Maibach). Articles remaining after screening underwent full text review, and articles meeting inclusion criteria were included. The data were extracted from the literature by one researcher (Maxwell Green) and verified by two others (Nadia Kashetsky, Aileen Feschuk). Any discrepancies in data collection were settled by a fourth researcher (Howard I. Maibach). The final data collected reported on significant effects of environmental factors and pollution on TEWL values.

## 4 RESULTS

### 4.1 Study characteristics

In total, 15 studies met all inclusion criteria and were therefore included in review (Table 1). Majority of studies (*n* = 11) researched effects of climate condition and seasonal variation including the following variables: seasonal variation (*n* = 5), indoor humidity (*n* = 2), daily variation, climate conditions, altitude, geographical location (*n* = 1 each). Additionally, four studies studied the impact of pollution on TEWL.

**TABLE 1:** Summary of environmental and pollution variables influencing transepidermal water loss (TEWL) values

Author (Year)	Variable	Sample size, male/female, nationality, age	Significant trend observed
Firooz et al.^15^	Daily changes in temperature and sun exposure	*n* = 12 N/A Iran Adults	No significant impact on TEWL from daily changes in temperature and sun exposure
Song et al.^16^	Season	*n* = 100 100/0 Korea Adults	Increased TEWL at the forehead in summer compared to winter No significant impact on TEWL between seasons at the cheek and forearm
Kim et al.^17^	Outdoor summer exposure	*n* = 20 0/20 Korea Adults	Increased TEWL at the forehead and forearm in outdoor summer conditions compared to indoor air‐conditioning
Wan et al.^18^	Season, temperature, humidity	*n* = 206 0/206 China Adults	Increased TEWL at the forehead during fall and winter compared to spring and summer Decreased TEWL with increasing temperature and humidity
Yang et al.^19^	Season	*n* = 100 0/100 China Adults	Increased TEWL at the cheek during fall and winter compared to spring and summer No significant impact on TEWL across seasons at the forearm
Doleckova et al.^20^	Season	*n* = 446 0/446 Czech Republic Adults	Decreased TEWL at the forehead during spring compared to fall, summer, and winter No significant impact on TEWL across seasons at the cheek
Jin et al.^21^	Indoor humidity	*n* = 22 NA Scotland Adults	No significant impact on TEWL across humidity levels
Jang et al.^22^	Indoor humidity	*n* = 11 0/11 Korea Adults	No significant impact on TEWL in less than 30% humidity or greater than 70% humidity
Liu et al.^23^	Atmospheric pressure, Temperature,Relative humidity, Steam pressure	*n* = 2005 N/A China Adults	Increased TEWL with increasing atmospheric pressure Decreased TEWL with increasing temperature, relative humidity, and steam pressure
Lee et al.^24^	Altitude	*n* = 136 0/136 Adults Indonesia	No significant impact on TEWL across altitudes
Mack et al.^25^	Geographical location	*n* = 495 N/A China, India, United States Children (*n* = 397), adults (*n* = 98))	Increased TEWL at the dorsal forearm and upper inner arm in Beijing children compared to children from Mumbai and New Jersey No significant impact on TEWL across adults between geographical regions
Shamsipour et al.^26^	Pollution: PM_10_, PM_2.5,_ CO, SO_2_, NO_2_, O_3_	*n* = 25 3/22 Iran Adults	Increased TEWL in the arm and forehead with increasing NO_2_ exposure
Kim et al.^27^	Pollution: PM	*n* = 100 0/100 China Adults	Increased TEWL in forearm of both older and younger women from the industrial region compared to the rural region No significant trend observed
Huang et al.^28^	Pollution: PM	*n* = 166 166/0 China Adults	Increased TEWL in the cheek of urban taxi drivers compared to rural taxi drivers
Oh et al.^29^	Pollution: PM	*n* = 25 0/25 Adults Korea	Increased TEWL during high PM exposure periods compared to low PM exposure periods in individuals

Abbreviations: CO, carbon monoxide; N/A, not available; NO_2_, nitrogen dioxide; O_3_, ozone; PM, particulate matter; SO_2_, sulphur dioxide; TEWL, transepidermal water loss.

### 4.2 Climate conditions and seasonal variation

#### 4.2.1 Seasonal variation in TEWL

Five studies examined seasonal variation on TEWL with mixed results. Overall, one study found higher TEWL values in skin during summer conditions compared to other months^16^ with one study showing increased TEWL in skin after being exposed to summer conditions.^17^ These results are contradicted by two studies that show increased TEWL in spring and winter months compared to summer and fall.^18^, ^19^ Finally, one study found TEWL of the forehead was significantly lower during spring months.^20^

First, Song et al.^16^ examined how skin integrity differed between summer and winter in healthy Korean males. All 100 subjects rested in a room 20–24°C with humidity from 40% to 60% for 30 min. A Tewameter TM300 was then used to make measurements at the forehead, cheek and forearm once in June to reflect summer values and once in January to reflect winter months. The average temperature and humidity in June were 24.9°C and 74.2% respectively. The average temperature and humidity in January were −2.4°C and 59.7% respectively. Results showed significantly higher TEWL values on the forehead during the summer (21.41 ± 4.99 g/h/m^2^) compared to the winter (17.40 ± 4.67 g/h/m^2^), and although summer averages for TEWL were higher at other sites, no significant difference was observed at the cheek and forearm.

Second, Kim et al.^17^ found similar results in a cohort of 20 Korean adult women. These subjects rested in a temperature and humidity controlled air‐conditioned environment before spending 20 min outside during the summer months shielded from direct sunlight. TEWL measurements were made at the forehead, forearm and cheek after rest in the air‐conditioning and after spending 20 min outside. Results showed significantly higher TEWL values in women at the forehead and forearm immediately spending time in the summer environment.

Wan et al.^18^ also examined seasonal variability in TEWL values but found contradictory results to those described above. They recruited 206 healthy Chinese women and measured TEWL values of the forehead during Spring (March–May), Summer (June–August), Fall (September–November), and Winter (December–February). Authors note that winter temperatures in this region remain above 10°C. Subjects rested for 30 min in a room 21 ± 1°C with humidity between 45% and 55% before each of the four measurements. A TM300 Tewameter was used to make measurements on the forehead of women at the same time of their menstrual cycle to minimise confounding variables. TEWL values in fall (17.4 ± 4.2 g/m^2^/h) and winter (18.2 ± 3.8 g/m^2^/h) were significantly higher than summer (9.9 ± 3.9 g/m^2^/h) and spring (13.3 ± 4.1 g/m^2^/h) with a significant negative association seen between TEWL and both temperature and humidity.

Yang et al.^19^ also studied Chinese females examining TEWL variation across seasons; 100 adult women rested for 20 min in a room between 20 and 25°C and 45%–55% humidity. TEWL measurements were made using a Tewameter on the right cheek and right forearm. Measurements were made three consecutive times on each woman on each of the four seasons, with the average being used for results. Results mirrored those found by Wan et al.,^18^ with higher TEWL values observed in the spring and winter compared to summer and fall on the cheek. No significant trend was observed across seasons at the forearm.

Doleckova et al.^20^ performed a similar study on Caucasian women, measuring TEWL during different seasons (Spring *n* = 85, Summer *n* = 93, Autumn *n* = 137, Winter *n* = 131). TEWL measurements were done after women rested for 30 min in a room 20–22°C with a humidity from 40 to 45; TEWL measurements were made on the forehead and cheek 10 consecutive times, with the average value being used for results. Results showed that TEWL values were significantly lower on the forehead during the spring (12.5 ± 2.6 g/h/m^2^) compared to summer (14.4 ± 3.5 g/h/m^2^), fall (14.1 ± 3.3 g/h/m^2^), and winter (14.2 ± 3.2 g/h/m^2^), with no significant difference seen at the cheek across seasons.

#### 4.2.2 Indoor humidity

Two studies examined effects of indoor humidity on TEWL. Overall, both studies showed no significant correlation between TEWL and relative humidity.

First, Jin et al.^21^ determined how indoor humidity affected skin integrity of both younger and older adults during winter months. They recruited 11 college students and 11 nursing home residents for the study. Both groups followed a typical daily routine, with college students attending class, and the nursing home residents attending activities in their facility. TEWL measurements were made using a Tewameter TM300 at the right volar forearm on four occasions at the dorm halls or nursing facility: a non‐intervened day between Feb 20 and Feb 26, an intervention with room humidity at 40%, a non‐intervened day between Mar 9 to Mar 12, and an intervention with room humidity at 50%. Results showed no significant correlation between relative humidity of the room and TEWL in either the young or older adults.

Second, Jang et al.^22^ also examined how humidity affects skin integrity in a cohort of 11 young women during sleep. The women first slept for greater than 7 h in a room with less than 30% humidity at 20 ± 2°; TEWL measurements were made using a Vapometer on the cheek of women, with another measurement being done after five consecutive tape‐strippings. These measurements were made before bed, immediately after waking, and after a face wash in the morning. The next night, the process was repeated in the same conditions at greater than 70% humidity. Results showed no significant difference in TEWL measurements between humidity levels. However, after a night in 30% humidity, TEWL was significantly increased after morning face‐wash compared to baseline. Overall, both studies showed no significant correlation between TEWL and relative humidity.

#### 4.2.3 Daily variation in TEWL

Firooz et al.^15^ examined if changes in sun exposure and temperature throughout a single day could lead to changes in TEWL within individuals. They recruited 12 healthy Iranian adults with Fitzpatrick skin types 3 and 4 for the study. Subjects rested in 22 ± 2°C room with humidity between 30% and 40% for 30 min before measurements were made each time. A TEWL metre made measurements at 8 am, 12 pm, and 4 pm on the right mid‐forearm. Subjects maintained standardised diets and social activities throughout the day and were to refrain from strenuous exercise. Results showed no significant difference between TEWL values at 8 am (3.97 ± 3.37 g/h/m^2^), 12 pm (4.57 ± 4.58 g/h/m^2^), or 4pm (3.17 ± 1.69 g/h/m^2^).

#### 4.2.4 Climate conditions

Liu et al.^23^ used a large cohort of 2005 Chinese volunteers to determine the influences of multiple climate conditions on TEWL values. Information on atmospheric pressure, temperature, steam pressure, and relative humidity was collected from local meteorologic stations and calculated the cumulative effects of each of these variables on TEWL. TEWL measurements were made on subjects in a room between 18 and 22°C with a humidity between 40% and 60% at six anatomical sites: forehead, cheek, nasolabial, inner forearm, dorsal hand, and palm. Results of multivariate linear regression showed a positive association between atmospheric pressure and TEWL and a negative association between temperature, steam pressure, and relative humidity and TEWL. At specific anatomic sites, one environmental factor affected TEWL the most within the regression model: temperature on the forehead, relative humidity on the forearm, steam pressure on the dorsal hand, and atmospheric pressure on the palm. These results indicate that the effects of climate variables may vary across anatomical regions.

#### 4.2.5 Altitude

Lee et al.^24^ looked at how different altitudes and their climate variation may impact TEWL in Indonesian females in Jakarta [low‐altitude (7 m)] and Bandung [high‐altitude (768 m)]. They reanalysed a preexisting dataset where TEWL baseline values were made in women of both regions on the forehead and cheek. Results showed no significant differences between the TEWL on the women from low altitude foreheads (13.4 g/m^2^/h) or cheeks (13.2 g/m^2^/h) compared to the foreheads (12.8 g/m^2^/h) and cheeks (14.6 g/m^2^/h) of women from high altitude.

#### 4.2.6 Geographical location

Mack et al.^25^ looked at how geography and ethnicity come together to affect TEWL in both adults and children across three sites in Beijing, Skillman, and Mumbai. There were 120 Chinese Children and 40 Chinese adults recruited in Beijing, 88 black children and 19 black adults recruited in Skillman, 84 white children and 20 white adults recruited in Skillman, and 105 Indian children and 40 Indian adults recruited in Mumbai. TEWL measurements were made on the dorsal forearm and upper inner arm on subjects after they rested in a temperature and humidity‐controlled room for 30 min. The average temperature during the study period for Beijing was 0°C, for Mumbai 29°C, and for Skillman 25°C. The average humidity for Beijing was 31%, for Mumbai 56%, and for Skillman 62%. Results showed no difference in TEWL in adults across all sites and ethnicities. TEWL values were higher at both the dorsal forearm and upper inner arm in Beijing children, an area with the lowest average temperature and humidity, compared to children from Mumbai or Skillman.

### 4.3 Pollution

Four studies analysed exposure to pollution and all concluded that exposure to either particulate matter or NO_2_ can increase TEWL. Overall, one study showed a positive significant relationship between nitrogen dioxide (NO_2_) exposure and TEWL, and three studies showed a significant relationship between particulate matter (PM) exposure and TEWL.

First, Shamsipour et al.^26^ recruited 25 participants aged 18–60 years old and followed them from April 2017 to April 2018. At six separate intervals across the study, a TEWAmeter was used to obtain TEWL measurements on subjects on the middle forehead and right upper arm. Then using local air quality data provided by Tehran monitoring stations, average daily exposure to various air pollutants was estimated for each person to calculate values of exposure for the 24 h prior to each of the six measurements or for multi‐day averages prior to measurements. The pollutants of interest were PM_10_, PM_2.5_, carbon monoxide (CO), sulphur dioxide (SO_2_), NO_2_, and ozone (O_3_). Results showed that exposure to NO_2_ showed a significant correlation with increased TEWL of the forehead in measurements 4, 5, and 6 and in the arm at measurements 1, 4, 5, and 6 based on linear models.

Kim et al.^27^ also looked at how chronic exposure to PM may negatively affect skin integrity in younger and older subjects. They recruited 50 younger women aged 25–35 years and 50 older women aged 55–65 years in an industrial region and compared them to an equivalent control population in Kunming, an area with much less pollution. TEWL measurements were made on the frontal cheek and inner forearm. Although no difference was shown in TEWL values of the cheek, both younger and older women in the industrial region showed significantly higher TEWL values compared to controls on the inner forearm. This suggests that chronic exposure to particulate matter may cause transient increases in TEWL values.

Similar results were observed by Huang et al.^28^ in a study conducted that compared urban taxi drivers to rural taxi drivers. Sixty‐six rural drivers from Chongming and 100 urban drivers from Shanghai between the ages of 28 and 55 who had been working as a driver for at least 5 years were recruited; after 1 h of the subjects resting in a testing room, TEWL measurements were made on the upper cheek using the Tewameter TM300. Five rounds of tape stripping on this same location were done using D‐squame tapes with 225 g/cm^3^ of pressure, and another TEWL measurement was done. Following 8 additional tape‐strippings, a final TEWL measurement was made. The average TEWL value of the upper cheek was significantly lower in rural drivers (16.5 ± 0.4 g/h/m^2^) compared to urban drivers (18.8 ± 0.5 g/h/m^2^). This difference was shown to only increase after the 13 rounds of tape stripping, supporting the conclusions drawn by the authors that skin exposed to pollution may be more sensitive to physical trauma.

Oh et al.^29^ also examined how PM exposure may affect TEWL values but compared values within 25 individuals after 14 days of high‐PM exposure and 14 days of low‐PM exposure. The high‐PM period occurred in the Spring of 2017 or 2018 based on local meteorological data, and the low‐PM period occurred during the summer months of either years. TEWL measurements were made on both cheeks of subjects at each period on days 1 and 14 after they had rested for 15–20 min in a temperature and humidity‐controlled room. Results showed significantly higher TEWL values at the cheeks during the high‐PM period (10.16 ± 4.77 g/m^2^/h) than the low‐PM period (5.99 ± 2.87 g/m^2^/h).

## 5 DISCUSSION

This review summarises the literature effects of climate and pollution on TEWL in humans after 2015 (*n* = 15 studies), providing an update to factors influencing TEWL in the literature by Akdeniz et al.^3^ and Honari and Maibach.^13^

Results for the effects of climate conditions and seasonal variation on TEWL values across 11 studies mirror mixed results literature prior to 2015. One study showed increased TEWL during summer months compared to winter,^16^ with one additional study showing that TEWL increased in women after they went from indoor air‐conditioned environments to outdoor summer weather.^17^ However, two additional studies then showed increased TEWL in facial skin during fall and winter months compared to summer and spring months with significant decreases in TEWL with increasing temperature.^18^, ^19^ There was some consensus across studies in regard to indoor relative humidity, though, with no significant trend in association with TEWL across two studies.

Discrepancies on the effects of temperature and relative humidity across seasons and geographic locations on TEWL suggest other variables may be affecting TEWL readings. Liu et al.^23^ also examined effects of atmospheric pressure and steam pressure and found that TEWL increases with increasing atmospheric pressure and decreases with increasing steam pressure. Very little research has looked more extensively into the effects of pressure in the atmosphere on skin integrity, but a study by Games et al^30^ showed that increasing local pressure leads to transient increases in skin temperature. This may suggest why increases in atmospheric pressure were linked to increases in TEWL, as increasing skin temperature has shown a significant linear relationship with increasing TEWL.^31^

The four studies studying the effect of pollution on TEWL all concluded that people residing in urban areas show higher TEWL values compared to those residing in rural areas and increasing TEWL values with increasing exposure to NO_2_ and PM. Exposure to PM in particular has been linked to skin diseases such as atopic dermatitis, potentially due to transient increases in TEWL.^32^ The mechanism of PM disturbances to skin integrity are not completely known, but they have been linked to epithelial oxidative stress and organelle dysfunction that can affect TEWL.^33^ Nitrogen dioxide may cause disruption to skin by similar mechanisms, but additional research on NO_2_ and other pollutants and how they affect barrier function is needed. Readers are directed to the *Handbook of Cosmetic Science and Technology 5* ^*th*^ *edition* for a comprehensive overview of how pollution can affect properties of the skin.

Several limitations of this review must be considered. Firstly, only studies published after 2015 were included; this decision was made so that this review may add to the already existing information presented in years prior. In addition, the search terms were created to capture a wide range of variables that may influence TEWL across anatomic regions, and thus, specific search criteria for each variable of interest were not used. In addition, this review only looked at human models (in vivo), while animal models may be beneficial in understanding climate and pollution effects on skin barrier function. The use of in vitro models may benefit research on TEWL changes due to environmental and pollution variation, as they allow for more controlled environmental conditions. Further, seasonal variation variables were prominently studied in Asian countries with temperatures not nearly as variable as many other climates across the world. This same limitation is observed with regards to altitude, with much greater variation in altitudes observed in many other countries. Also, heterogeneity in patient populations between studies makes direct comparison of the variable's impact on TEWL difficult. Finally, it is important to note that a statistically significant mathematical difference in TEWL values may not reflect differences in clinical presentation of the skin itself, and although significant differences may be shown in studies, many uncontrolled for variables likely impacted the differences in TEWL between individuals.

## 6 CONCLUSION

Effects of climate and seasonal variation on TEWL are due to a wide range of variables and their interactions that cannot be exclusively accounted for by temperature and relative humidity. This is demonstrated by the large number of contradictions found between temperature, relative humidity, and seasonal variation and their effects on TEWL. Additional studies should focus on how climate conditions other than temperature and humidity, such as atmospheric and steam pressure, may impact TEWL. In addition, it is important to control for variables affecting TEWL in experimental settings that have been supported by the literature such as age and anatomic location to minimise confounding in TEWL measurement.^5^

Pollution from particulate matter and NO_2_ cause oxidative damage to epithelial cells to increase TEWL as concluded by four studies in this review. This may put individuals living in industrial zones at higher risk for conditions of impaired barrier function such as atopic dermatitis and psoriasis. Additional research is needed to understand how other air pollutants may disrupt barrier function to gain a greater understanding of the mechanisms behind pollutant‐induced TEWL increases.

## CONFLICT OF INTEREST

None to declare.

## AUTHOR CONTRIBUTIONS

**Maxwell Green:** data curation, formal analysis, investigation, methodology, project administration, writing – original draft, writing – review & editing. **Nadia Kashetsky:** Data curation, formal analysis, investigation, methodology, writing – original draft, writing – review & editing. **Aileen Feschuk:** Data curation, formal analysis, investigation, methodology, project administration, writing – original draft, writing – review & editing. **Howard Maibach:** Conceptualization, formal analysis, investigation, project administration, supervision, writing – review & editing.

## Data Availability

This review did not create any new data sets, so no data availability statement is applicable.
